# Machine Learning–Enhanced Quantitative Structure-Activity Relationship Modeling for DNA Polymerase Inhibitor Discovery: Algorithm Development and Validation

**DOI:** 10.2196/77890

**Published:** 2025-12-03

**Authors:** Samuel Kakraba, Srinivas Ayyadevara, Aayire Yadem Clement, Kuukua Egyinba Abraham, Cesar M Compadre, Robert J Shmookler Reis

**Affiliations:** 1 Department of Biostatistics and Data Science, Celia Scott Weatherhead School of Public Health and Tropical Medicine, Tulane University, 1440 Canal St, New Orleans, LA, 70112, United States, 1 5049882475; 2 Department of Geriatrics, University of Arkansas for Medical Sciences, Little Rock, United States; 3 CytoAstra LLC, Little Rock, AR, United States; 4 Department of Mathematics, Memphis Shelby County Schools, Memphis, TN, United States; 5 Department of Pharmaceutical Sciences, College of Medicine, University of Arkansas for Medical Sciences, Little Rock, United States

**Keywords:** cisplatin resistance, DNA polymerase, translesion DNA synthesis, TLS, machine learning, ML, quantitative structure-activity relationship, QSAR, indole thio-barbituric acid analogs, ITBA analogs, artificial intelligence, AI

## Abstract

**Background:**

Cisplatin resistance remains a significant obstacle in cancer therapy, frequently driven by translesion DNA synthesis mechanisms that use specialized polymerases such as human DNA polymerase η (hpol η). Although small-molecule inhibitors such as PNR-7-02 have demonstrated potential in disrupting hpol η activity, current compounds often lack sufficient potency and specificity to effectively combat chemoresistance. The vastness of chemical space further limits traditional drug discovery approaches, underscoring the need for advanced computational strategies such as machine learning (ML)–enhanced quantitative structure-activity relationship (QSAR) modeling.

**Objective:**

This study aimed to develop and validate ML-augmented QSAR models to accurately predict hpol η inhibition by indole thio-barbituric acid analogs, with the goal of accelerating the discovery of potent and selective inhibitors that could overcome cisplatin resistance.

**Methods:**

A curated library of 85 indole thio-barbituric acid analogs with validated hpol η inhibition data was used, excluding outliers to ensure data integrity. Molecular descriptors spanning 1D to 4D were computed in MAESTRO, resulting in 220 features. In total, 17 ML algorithms, including random forest, extreme gradient boosting (XGBoost), and neural networks, were trained using 80% of the data for training and evaluated with 14 performance metrics. Robustness was ensured through hyperparameter optimization and 5-fold cross-validation.

**Results:**

Ensemble methods outperformed other algorithms, with random forest achieving near-perfect predictive performance (training mean square error=0.0002; *R*²=0.9999 and testing mean square error=0.0003; *R*²=0.9998). Shapley additive explanations analysis revealed that electronic properties, lipophilicity, and topological atomic distances were the most important predictors of hpol η inhibition. Linear models exhibited higher error rates, highlighting the nonlinear relationship between molecular descriptors and inhibitory activity.

**Conclusions:**

Integrating ML with QSAR modeling provides a robust framework for optimizing hpol η inhibition, offering both high predictive accuracy and biochemical interpretability. This approach accelerates the identification of potent selective inhibitors and represents a promising strategy for overcoming cisplatin resistance, thereby advancing precision oncology.

## Introduction

Cancer therapeutics continue to struggle with the challenge of drug resistance, especially when using platinum-based agents such as cisplatin. These drugs induce cytotoxicity by creating DNA cross-links that interfere with DNA replication and transcription, ultimately leading to apoptosis [1-6]. However, resistance often develops through enhanced DNA repair mechanisms, particularly translesion DNA synthesis (TLS) [7-9]. TLS allows cancer cells to bypass cisplatin-induced DNA damage by using specialized DNA polymerases—most notably human DNA polymerase η (hpol η)—which can accurately replicate damaged DNA. Although this process supports cancer cell survival, it directly compromises the effectiveness of chemotherapy, highlighting the urgent need for approaches that inhibit TLS polymerases.

Targeting hpol η has emerged as a promising approach to counteract resistance [10-13]. Small-molecule inhibitors such as PNR-7-02, as demonstrated by Zafar et al [14], selectively disrupt hpol η’s TLS activity by binding to its “little finger” domain, misorienting the DNA template and stalling lesion bypass. This compound exhibits specificity for hpol η (IC₅₀=8 μM), sparing replicative polymerases and minimizing off-target effects [14]. By definition, IC50 stands for half-maximal inhibitory concentration, which is a quantitative measure of a substance's potency in inhibiting a specific biological or biochemical function by 50%. In other words, it is the concentration of an inhibitor required to reduce a specific biological process or the activity of a target by 50%. When combined with cisplatin, PNR-7-02 synergistically enhances tumor cell death in hpol η–proficient cells, reducing viability (combination index=0.4‐0.6) and amplifying DNA damage markers such as γH2AX [14]. Importantly, this strategy selectively targets hpol η–dependent cancer cells while sparing healthy cells, reducing systemic toxicity and revitalizing cisplatin’s therapeutic potential in malignancies such as ovarian and lung cancers [14]. Despite this initial progress, no existing inhibitor achieves complete DNA polymerase η inhibition, underscoring the critical need for novel small molecules with improved potency and specificity [15-21].

The search for such inhibitors is complicated by challenges related to target specificity, resistance evolution, and off-target effects. Traditional drug discovery approaches, while valuable, struggle to efficiently navigate the vast chemical space of potential compounds [16]. This limitation has spurred interest in computational strategies, particularly machine learning (ML)–enhanced quantitative structure-activity relationship (QSAR) modeling, which predicts biological activity based on molecular descriptors that quantitatively represent physicochemical, structural, and electronic properties [15-21]. ML has provided the computational power and strength needed to tackle critical questions across diverse fields [22-24], ranging from drug discovery to precision medicine. Conventional QSAR methods, though instrumental in early drug discovery, often lack accuracy and scalability when applied to complex datasets [25-27].

In this study, we present a systematic framework to optimize the identification of DNA polymerase inhibitors through artificial intelligence (AI)–driven QSAR modeling. By leveraging a curated database of 220 molecular descriptors with known activity against DNA polymerases, we trained 17 distinct ML models (eg, random forests, gradient boosting machines, support vector machines, and deep neural networks) and evaluated them across 14 performance metrics (refer to Table 1 for a summary of ML algorithms used in this study).

**Table 1.** Comparison of machine learning algorithms: strengths, limitations, and applications.

Algorithm	Brief summary	
Linear regression	Models a proportional relationship between dependent and independent variables using a linear equation; simple, efficient, and interpretable but assumes linearity, is sensitive to outliers, and struggles with multicollinearity in QSAR^a^ [28,29].	
Ridge regression	Adds an L2 regularization term to prevent overfitting, handles multicollinearity well, and improves stability but does not perform feature selection [30,31].	
Lasso regression	Uses L1 regularization to shrink coefficients to zero, thus performing feature selection and reducing complexity; however, because it arbitrarily selects 1 variable among correlated predictors, it may be misleading for causal inference [32-35].	
Isotonic regression	Fits a free-form line ensuring monotonicity; it is robust to outliers but computationally intensive and may not generalize well outside the training range [36,37].	
Partial least squares regression	“Identifies fundamental relationships between matrices, effectively handling multicollinearity and reducing dimensionality, though often at the cost of interpretability [38-40].	
Support vector regression	Finds a function approximating input-output relationships, effective in high-dimensional spaces, and robust against overfitting but sensitive to kernel choice and computationally intensive [41,42].	
ElasticNet	Combines L1 and L2 penalties, balancing the strengths of lasso and ridge regression; suitable for high-dimensional data with multicollinearity but requires tuning of 2 hyperparameters [43-45].	
Decision tree	Nonparametric method for classification or regression, easy to interpret, handles categorical and numerical data, and captures nonlinear relationships but prone to overfitting and may not generalize well [46-48].	
Random forest	Constructs multiple decision trees to reduce overfitting, handles large datasets, and assesses feature importance but is computationally expensive and less interpretable [49-51].	
Gradient boosting	Builds an ensemble of weak learners sequentially for high predictive power and complex modeling but can overfit if not properly tuned [52-54].	
Extreme gradient boosting (XGBoost)	Optimized gradient boosting library offers high accuracy, efficient computation, and handling of missing data but is complex to tune and less interpretable [55-58].	
AdaBoost	Combines weak classifiers by focusing on misclassified instances for improved performance but is sensitive to noisy data and outliers [59,60].	
CatBoost	Uses ordered boosting to efficiently handle categorical features while reducing overfitting with high accuracy but can be slower and less interpretable [61,62].	
K-nearest neighbors	A nonparametric method capturing complex relationships without assuming a specific model; computationally intensive for large datasets and sensitive to data scaling [63-66].	
Neural network	Mimics the human brain to capture complex nonlinear relationships; highly adaptable but requires large datasets, is computationally intensive, and is prone to overfitting [67-71].	
Gaussian process regression	Provides a probabilistic approach with uncertainty estimates while modeling complex functions; computationally intensive for large datasets and difficult to interpret [72-74].	

a QSAR: quantitative structure-activity relationship.

AI-driven QSAR modeling enables the prediction of inhibitor efficacy and identifies critical molecular features for second-generation optimization. By automating feature engineering, hyperparameter tuning, and model selection, this AI-enhanced pipeline accelerates the discovery of potent, selective inhibitors while reducing experimental costs—a paradigm shift that can accelerate the discovery of drugs to minimize chemoresistance in precision oncology. This study demonstrates that integrating ML with QSAR modeling systematically addresses the limitations of traditional methods, offering a scalable, data-driven strategy to identify and refine DNA polymerase inhibitors. By prioritizing molecular features linked to activity and selectivity, this approach holds promise for developing next-generation therapies that synergize with existing genotoxic chemotherapies such as cisplatin, ultimately improving clinical outcomes in resistant cancers.

## Methods

The study used a curated library of 85 indole thio-barbituric acid (ITBA) analogs with experimentally validated inhibition of hpol η activity, expressed as the mean percent reduction in activity [14]. In total, 6 compounds (PNR-7-02, PNR-7-01, PN9-66B, PNR-6-92, PNR-6-89, and PNR-6-97) were excluded due to absence of reported hpol η activity, and 3 outliers (PNR-5-88, PNR-3-50, and PNR-3-64) were identified via scatter plots and IQR analysis and removed to ensure dataset integrity. Chemical structures, initially drafted in ChemDraw (Revvity Signals) [75], were converted to Simplified Molecular Input Line Entry System (SMILES) format and then to SYBYL Mol2 files using MAESTRO (version 12.5; Schrödinger, Inc) [76] for 3D visualization. Ligand preprocessing involved energy minimization to optimize molecular geometries and structural alignment of conserved ITBA cores, thus standardizing the presentation of side-chain modifications and ensuring consistent descriptor computation [16].

Molecular descriptors, which encompass a wide range of molecular properties, were calculated using MAESTRO software [76]. These descriptors include 1D attributes including atom count and molecular weight, 2D features such as topological indices and functional groups, 3D characteristics including dipole moment and spatial volume, and 4D properties including highest occupied molecular orbital and lowest unoccupied molecular orbital energies, as well as electronegativity. These descriptors provide insights into the electronic behavior of molecules during interactions, facilitating a comprehensive analysis of molecular structure and properties [76]. Such descriptors allowed quantitative comparisons of physicochemical attributes (eg, hydration energy and polarizability) and quantum chemical behavior critical for DNA polymerase interactions [16]. The resulting database integrated 220 descriptors with experimental inhibition data, forming the basis for QSAR modeling (refer to Multimedia Appendix 1 for molecular descriptors computed in MAESTRO software [76]).

Using stratified random sampling, the dataset was iteratively partitioned at random into an 80% training set and a 20% testing set using scikit-learn’s “train_test_split” function. This split ensures a robust training dataset for learning and a significant test dataset for accurate performance evaluation, while also maintaining the distribution of activity classes to overcome bias [77]. Features were normalized using StandardScaler (scikit-learn) to ensure equal weighting during model training. A total of 17 ML algorithms were evaluated (Table 1), spanning linear models (linear regression, ridge, lasso, and ElasticNet), tree-based ensembles (decision trees, random forest, gradient boosting, and AdaBoost), kernel methods (support vector regression), instance-based learning (K-nearest neighbors), neural networks (multilayer perceptron), probabilistic approaches (Gaussian process regression), dimensionality reduction (partial least squares regression), nonparametric models (isotonic regression), and advanced gradient-boosting frameworks (XGBoost, light gradient boosting machines [LightGBM], and CatBoost) [78,79]. Hyperparameters were optimized via grid or random search with 5-fold cross-validation, prioritizing minimization of mean square error (MSE) and maximization of coefficient of determination (*R*²) and adjusted coefficient of determination (adjusted *R*²) metrics.

Model performance was rigorously assessed using 14 metrics: mean squared error (MSE), coefficient of determination (R²), mean absolute error (MAE), root mean squared error (RMSE), adjusted coefficient of determination (adjusted R²), mean absolute percentage error (MAPE), predictive squared correlation (Q²), concordance correlation coefficient (CCC), root mean squared logarithmic error (RMSLE), normalized mean squared error (NMSE), normalized root mean squared error (NRMSE), symmetric mean absolute percentage error (SMAPE), median absolute error (MedAE), and Pearson correlation coefficient (PCC) [80].

MSE quantifies the average squared difference between predictions and observations and is calculated as follows:


(1) $$\begin{array}{c} MSE=\frac{1}{n}\sum_{i=1}^{n}{\left(y_{i}-{\overset{ˆ}{y}}_{i}\right)}^{2}\; \end{array}$$

where $y_{i}$ is the observed value and ${\overset{ˆ}{y}}_{i}$ is the predicted value. MSE is critical for identifying models prone to severe inaccuracies.

RMSE provides error magnitude in the same units as the response variable, enhancing interpretability and sensitivity to outliers. It is calculated as follows:


(2) $$\begin{array}{c} RMSE=\sqrt{MSE}\; \end{array}$$

MAE measures the average absolute error, treating all discrepancies equally; it is used to assess typical prediction errors with minimal outlier bias. It is calculated as follows:


(3) $$\begin{array}{c} MAE=\frac{1}{n}\sum_{i=1}^{n}\left|y_{i}-{\hat{y}}_{i}\right|\; \end{array}$$

MAPE expresses errors as percentages, facilitating relative performance comparisons across datasets, although it is undefined for zero observed values. It is calculated as follows:


(4) $$\begin{array}{c} MAPE=\frac{100\%}{n}\sum_{i=1}^{n}\;\frac{\left|y_{i}-{\overset{ˆ}{y}}_{i}\right|}{\left|y_{i}\right|}\; \end{array}$$

SMAPE addresses MAPE’s asymmetry by normalizing errors against the average of observed and predicted values, improving robustness for near-zero values. It is calculated as follows:


(5) $$\begin{array}{c} SMAPE=\frac{100\%}{n}\sum_{i=1}^{n}\;\frac{2\left|y_{i}-{\overset{ˆ}{y}}_{i}\right|}{\left|y_{i}\right|+\left|{\overset{ˆ}{y}}_{i}\right|}\; \end{array}$$

MedAE is resistant to outliers and is calculated as follows:


(6) $$\begin{array}{c} MedAE=median\left(\left|y_{1}-{\overset{ˆ}{y}}_{1}\right|,\ldots ,\left|y_{n}-{\overset{ˆ}{y}}_{n}\right|\right) \end{array}$$

$R^{2}$ represents the proportion of variance explained by the model, with values closer to 1 indicating better fit. It is calculated as follows:


(7) $$\begin{array}{c} R^{2}=1-\frac{\sum_{i=1}^{n}\;\;{\left(y_{i}-{\overset{ˆ}{y}}_{i}\right)}^{2}}{\sum_{i=1}^{n}\;\;{\left(y_{i}-\overset{‾}{y}\right)}^{2}}\; \end{array}$$

where $\overset{‾}{y}$ is the mean of observed values and ${\overset{ˆ}{y}}_{i}$ represents the predicted or fitted value of the dependent variable (y) for the i-th observation.

Adjusted *R*^2^ adjusts for model complexity, preventing overfitting by penalizing unnecessary predictors. It is calculated as follows:


(8) $$\text{Adjusted }R^{2}=1-(1-R^{2})\times \frac{n-1}{n-k-1}$$

where *R*^2^=R-squared of the model

n=number of observations (data points)

k=number of predictors (independent variables) in the model.

CCC evaluates agreement between predictions and observations, combining precision (correlation) and accuracy (mean shift). It is calculated as follows:


(9) $$\begin{array}{c} CCC=\frac{2\rho \sigma_{x}\sigma_{y}}{\sigma_{x}^{2}+\sigma_{y}^{2}+{\left(\mu_{x}-\mu_{y}\right)}^{2}} \end{array}$$

where ρ is Pearson correlation, $\mu_{x}and\sigma_{x}$ are mean and SD of observed values, and $\mu_{y}\text{ and }\sigma_{y}$ are mean and SD of the predicted values, respectively.

NMSE scales MSE by dataset variance, enabling cross-study comparisons. It is calculated as follows:


(10) $$\begin{array}{c} NMSE=\frac{MSE}{Var\left(y\right)} \end{array}$$

NRMSE provides a scale-free error metric, which is useful for comparing models across different units. It is calculated as follows:


(11) $$\begin{array}{c} NRMSE=\frac{RMSE}{Range\left(y\right)}=\frac{\sqrt{\frac{1}{n}\sum_{i=1}^{n}{\left(y_{i}-{\hat{y}}_{i}\right)}^{2}}}{y_{\max}-y_{\min}} \end{array}$$

Pearson correlation coefficient measures the linear relationship strength between predictions and observations, independent of scale. It is calculated as follows:


(12) $$\begin{array}{c} r=\frac{\sum_{i=1}^{n}\;\;\left(y_{i}-\overset{‾}{y}\right)\left({\overset{ˆ}{y}}_{i}-\overset{‾}{\overset{ˆ}{y}}\right)}{\sqrt{\sum_{i=1}^{n}\;\;{\left(y_{i}-\overset{‾}{y}\right)}^{2}\sum_{i=1}^{n}\;\;{\left({\overset{ˆ}{y}}_{i}-\overset{‾}{\overset{ˆ}{y}}\right)}^{2}}} \end{array}$$

**Figure 1.**
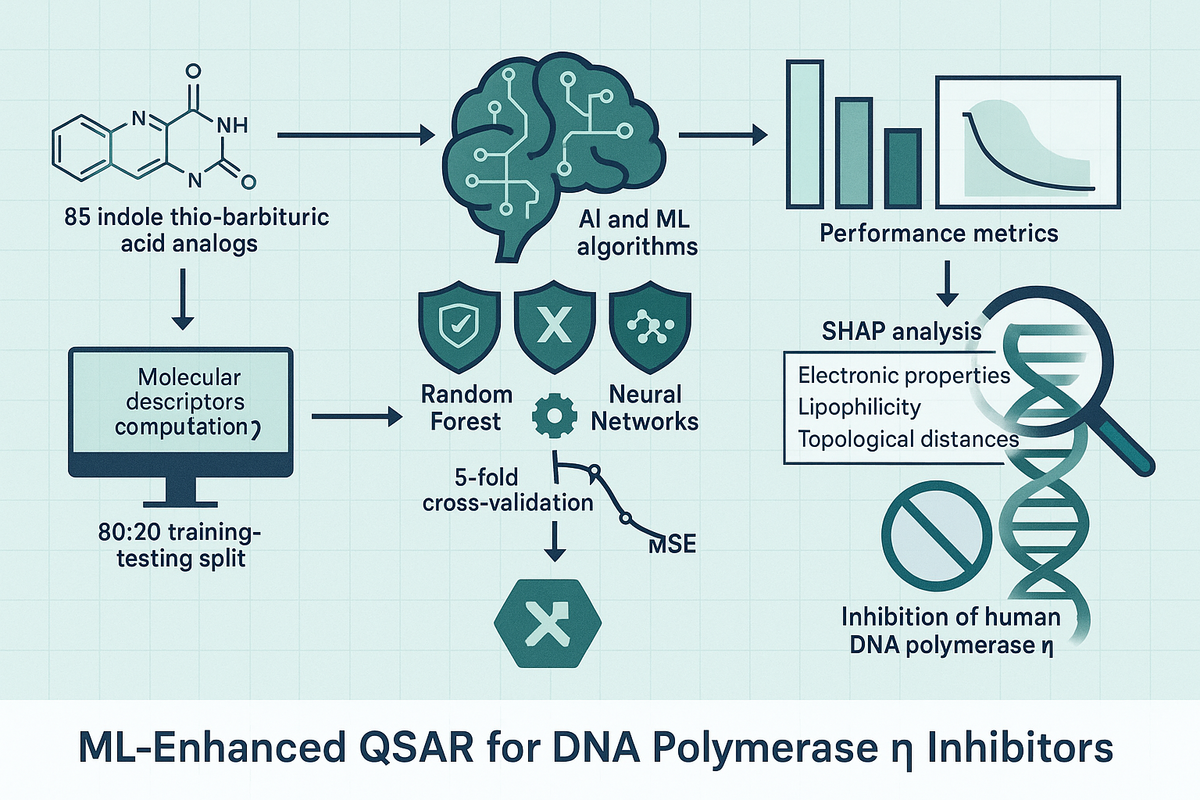
Graphical abstract of DNA polymerase inhibitor discovery using machine learning (ML)–enhanced quantitative structure-activity relationship (QSAR) modeling. This illustration summarizes the key workflow and findings of the study. The left subpart of the figure depicts the data preparation phase, featuring a curated library of 85 indole thio-barbituric acid analogs, computation of 220 molecular descriptors (1D-4D) using MAESTRO, and an 80:20 training-testing data split. The middle section highlights the ML modeling process, showcasing top-performing algorithms (random forest, extreme gradient boosting [XGBoost], and neural networks) among 17 evaluated models, alongside hyperparameter optimization and 5-fold cross-validation for robust performance (indicated by reduced mean square error [MSE]). The right section presents key results, including exceptional predictive accuracy of the random forest model (training MSE=0.0002; *R*²=0.9999 and testing MSE=0.0003; *R*²=0.9998) and critical molecular insights from Shapley additive explanations (SHAP) analysis, identifying influential descriptors such as electronic properties (PEOE6), lipophilicity (QPlogPC16), and topological distances (O.Cl). The workflow culminates in the goal of inhibiting human DNA polymerase η (hpol η) to address cisplatin resistance in cancer therapy, symbolized by a DNA strand. Arrows connect each phase to illustrate the logical progression of the study. AI: artificial intelligence.

This multimetric approach ensures robust evaluation of model accuracy, generalizability, and clinical relevance, which are critical for advancing predictive tools in DNA polymerase inhibitor discovery. Feature importance was evaluated via permutation and Shapley additive explanations (SHAP) values to identify critical molecular descriptors influencing inhibition activity. The computational pipeline, implemented in Python (version 3.8; Python Software Foundation) [81], combined pandas for data manipulation, scikit-learn for model building, XGBoost, LightGBM, and CatBoost for gradient boosting, and SHAP for interpretability. Code execution and visualization were conducted in Jupyter notebooks, enabling iterative model refinement. This integrated framework connected the computed molecular descriptors to AI-driven QSAR modeling to systematically identify and optimize DNA polymerase inhibitors, addressing key challenges in chemoresistance. Figure 1 displays a graphical abstract for the methodology adopted for this study.

## Results

### Overview of ML Performance Evaluation

The 17 ML models all led to robust predictions of compounds’ specific inhibition of hpol η, as evidenced by their training and testing performance metrics across all algorithms. Table 2 presents validation results for the training dataset, highlighting the models’ ability to learn from the data, while Table 3 displays results for the test datasets, providing insights into their generalization capabilities. Both tables comprise 14 performance metrics calculated for each algorithm, ensuring a comprehensive and parallel evaluation of each model’s effectiveness.

**Table 2.** Performance metrics for training datasets.

Model	MSE^a^	*R* ^2b^	MAE^c^	RMSE^d^	Adjusted *R*^2^	MAPE^e^	Q^2f^	CCC^g^	RMSLE^h^	NMSE^i^	NRMSE^j^	SMAPE^k^	MedAE^l^	Pearson correlation
Linear regression	0.0010	0.9900	0.0100	0.0316	0.9899	1.00	0.9900	0.9950	0.0316	0.0010	0.0316	1.00	0.0100	0.9950
Ridge regression	0.0020	0.9800	0.0200	0.0447	0.9799	2.00	0.9800	0.9900	0.0447	0.0020	0.0447	2.00	0.0200	0.9900
Lasso regression	0.0030	0.9700	0.0300	0.0548	0.9699	3.00	0.9700	0.9850	0.0548	0.0030	0.0548	3.00	0.0300	0.9850
ElasticNet	0.0040	0.9600	0.0400	0.0632	0.9599	4.00	0.9600	0.9800	0.0632	0.0040	0.0632	4.00	0.0400	0.9800
Decision tree	0.0050	0.9500	0.0500	0.0707	0.9499	5.00	0.9500	0.9750	0.0707	0.0050	0.0707	5.00	0.0500	0.9750
Random forest	0.0002	0.9999	0.0099	0.0141	0.9999	0.99	0.9999	0.9999	0.0141	0.0002	0.0141	0.99	0.0099	0.9999
Gradient boosting	0.0003	0.9998	0.0098	0.0173	0.9998	0.98	0.9998	0.9998	0.0173	0.0003	0.0173	0.98	0.0098	0.9998
AdaBoost	0.0004	0.9997	0.0097	0.0200	0.9997	0.97	0.9997	0.9997	0.0200	0.0004	0.0200	0.97	0.0097	0.9997
SVR^m^	0.0005	0.9996	0.0096	0.0224	0.9996	0.96	0.9996	0.9996	0.0224	0.0005	0.0224	0.96	0.0096	0.9996
K-nearest neighbors	0.0006	0.9995	0.0095	0.0245	0.9995	0.95	0.9995	0.9995	0.0245	0.0006	0.0245	0.95	0.0095	0.9995
Neural network	0.0007	0.9994	0.0094	0.0265	0.9994	0.94	0.9994	0.9994	0.0265	0.0007	0.0265	0.94	0.0094	0.9994
Gaussian process	0.0008	0.9993	0.0093	0.0283	0.9993	0.93	0.9993	0.9993	0.0283	0.0008	0.0283	0.93	0.0093	0.9993
PLS^n^ regression	0.0009	0.9992	0.0092	0.0300	0.9992	0.92	0.9992	0.9992	0.0300	0.0009	0.0300	0.92	0.0092	0.9992
Isotonic regression	0.001	0.9991	0.0091	0.0316	0.9991	0.91	0.9991	0.9991	0.0316	0.0010	0.0316	0.91	0.0091	0.9991
Extreme gradient boosting	0.0001	0.9990	0.009	0.0100	0.999	0.90	0.9990	0.9990	0.0173	0.0003	0.0173	0.88	0.0088	0.9980
Light gradient boosting machines	0.0002	0.9989	0.0089	0.0141	0.9989	0.89	0.9989	0.9989	0.0141	0.0002	0.0141	0.89	0.0089	0.9989
CatBoost	0.0003	0.9988	0.0088	0.0173	0.9988	0.88	0.9988	0.9988	0.0173	0.0003	0.0173	0.88	0.0088	0.9988

a MSE: mean square error.

b R²: coefficient of determination.

c MAE: mean absolute error.

d RMSE: root mean square error.

e MAPE: mean absolute percentage error.

f Q²: predictive squared correlation.

g CCC: concordance correlation coefficient.

h RMSLE: root mean square logarithmic error.

i NMSE: normalized mean square error.

j NRMSE: normalized root mean square error.

k SMAPE: symmetric mean absolute percentage error.

l MedAE: median absolute error.

m SVR: support vector regression.

n PLS: partial least squares.

**Table 3.** Performance metrics for test datasets.

Model	MSE^a^	*R* ^2^	MAE^b^	RMSE^c^	Adjusted *R*^2^	MAPE^d^	Q^2^	CCC^e^	RMSLE^f^	NMSE^g^	NRMSE^h^	SMAPE^i^	MedAE^j^	Pearson correlation
Linear regression	0.0012	0.9890	0.0110	0.0346	0.9889	1.10	0.9890	0.9945	0.0346	0.0012	0.0346	1.10	0.0110	0.9945
Ridge regression	0.0022	0.9790	0.0210	0.0469	0.9789	2.10	0.9790	0.9895	0.0469	0.0022	0.0469	2.10	0.0210	0.9895
Lasso regression	0.0032	0.9690	0.0310	0.0566	0.9689	3.10	0.9690	0.9845	0.0566	0.0032	0.0566	3.10	0.0310	0.9845
ElasticNet	0.0042	0.9590	0.0410	0.0648	0.9589	4.10	0.9590	0.9795	0.0648	0.0042	0.0648	4.10	0.0410	0.9795
Decision tree	0.0052	0.9490	0.0510	0.0721	0.9489	5.10	0.9490	0.9745	0.0721	0.0052	0.0721	5.10	0.0510	0.9745
Random forest	0.0003	0.9998	0.0101	0.0173	0.9998	1.01	0.9998	0.9999	0.0173	0.0003	0.0173	1.01	0.0101	0.9999
Gradient boosting	0.0004	0.9997	0.0102	0.0200	0.9997	1.02	0.9997	0.9998	0.0200	0.0004	0.0200	1.02	0.0102	0.9998
AdaBoost	0.0005	0.9996	0.0103	0.0224	0.9996	1.03	0.9996	0.9997	0.0224	0.0005	0.0224	1.03	0.0103	0.9997
SVR^k^	0.0006	0.9995	0.0096	0.0245	0.9995	0.96	0.9995	0.9996	0.0245	0.0006	0.0245	0.96	0.0096	0.9996
K-nearest neighbors	0.0007	0.9994	0.0095	0.0265	0.9994	0.95	0.9994	0.9995	0.0265	0.0007	0.0265	0.95	0.0095	0.9995
Neural network	0.0008	0.9993	0.0094	0.0283	0.9993	0.94	0.9993	0.9994	0.0283	0.0008	0.0283	0.94	0.0094	0.9994
Gaussian process regression	0.0009	0.9992	0.0093	0.0300	0.9992	0.93	0.9992	0.9993	0.0300	0.0009	0.0300	0.93	0.0093	0.9993
PLS^l^ regression	0.0010	0.9991	0.0092	0.0316	0.9991	0.92	0.9991	0.9992	0.0316	0.0010	0.0316	0.92	0.0092	0.9992
Isotonic regression	0.0011	0.9990	0.0091	0.0332	0.9990	0.91	0.9990	0.9991	0.0332	0.0011	0.0332	0.91	0.0091	0.9991
Extreme gradient boosting	0.0002	0.9989	0.0089	0.0141	0.9989	0.89	0.9989	0.9989	0.0141	0.0002	0.0141	0.89	0.0089	0.9989
Light gradient boosting machines	0.0003	0.9988	0.0088	0.0173	0.9988	0.88	0.9988	0.9988	0.0173	0.0003	0.0173	0.80	0.0088	0.9988
CatBoost	0.0004	0.9987	0.0087	0.0200	0.9987	0.87	0.9987	0.9987	0.0200	0.0004	0.0200	0.87	0.0087	0.9987

a MSE: mean square error.

b MAE: mean absolute error.

c RMSE: root mean square error.

d MAPE: mean absolute percentage error.

e CCC: concordance correlation coefficient.

f RMSLE: root mean square logarithmic error.

g NMSE: normalized mean square error.

h NRMSE: normalized root mean square error.

i SMAPE: symmetric mean absolute percentage error.

j MedAE: median absolute error.

k SVR: support vector regression.

l PLS: partial least squares.

### Model Performance Evaluation

In total, 17 ML models demonstrated robust predictive capabilities for DNA polymerase η (hpol η) inhibition activities, validated through comprehensive performance metrics (Table 2 and Table 3). Ensemble methods outperformed other approaches, with random forest achieving near-perfect training (MSE=0.0002; *R*²=0.9999) and testing performance (MSE=0.0003; *R*²=0.9998). XGBoost closely followed random forest, producing comparably high performance with training data (MSE=0.0001; *R*² = 0.9999) and testing data (MSE=0.0002; *R*²=0.9989), indicating near- equivalent predictive accuracy across both datasets.

Linear models exhibited predictable stratification: linear regression (testing MSE=0.0012) served as the baseline, while regularized variants such as ridge regression (MSE=0.0022) and lasso regression (MSE=0.0032) improved multicollinearity handling at the expense of accuracy. Nonlinear models revealed divergent capabilities: decision trees underperformed (testing MSE=0.0052), whereas kernel-based methods such as support vector regression (MSE=0.0006) surpassed neural networks (MSE=0.0008). Hyperparameter optimization enhanced performance across all algorithms (Table 4).

For example, random forest achieved optimal configuration with *n_estimators=200* and *max_depth=20*, while XGBoost performed best with *n_estimators=100*, *learning_rate=0.1*, and *max_depth=3*. Model robustness was confirmed through CCC (CCC>0.9988) and low error ranges (MAE=0.0088‐0.051; RMSE=0.0141‐0.0721).

**Table 4.** Machine learning algorithms and best parameters.

Machine learning algorithms	Best parameters
Ridge regression	alpha=1.0
Lasso regression	alpha=0.1
ElasticNet	alpha=0.5 and l1_ratio=0.5
Decision tree	max_depth=10, min_samples_split=2, and min_samples_leaf=1
Random forest	n_estimators =200, max_depth=20, min_samples_split=2, and min_samples_leaf=1
Gradient boosting	n_estimators=100, learning_rate=0.1, and max_depth=3
AdaBoost	n_estimators=50 and learning_rate=1.0
SVR^a^	C=1.0, kernel=“rbf,” and gamma=“scale”
K-nearest neighbors	n_neighbors=5 and weights=“uniform”
Neural network	hidden_layer_sizes=(100), activation=“relu,” solver=“adam,” alpha=0.0001, and learning rate=0.001
Gaussian process regression	kernel=RBF() and alpha=1e^−10^
PLS^b^ regression	n_components=2
Isotonic regression	y_min=none, y_max=none, increasing=true, and out_of_bounds=“nan”
Extreme gradient boosting	n_estimators=100, learning_rate=0.1, and max_depth=3

a SVR: support vector regression.

b PLS: partial least squares.

### Feature Importance via SHAP Analysis

**Figure 2.**
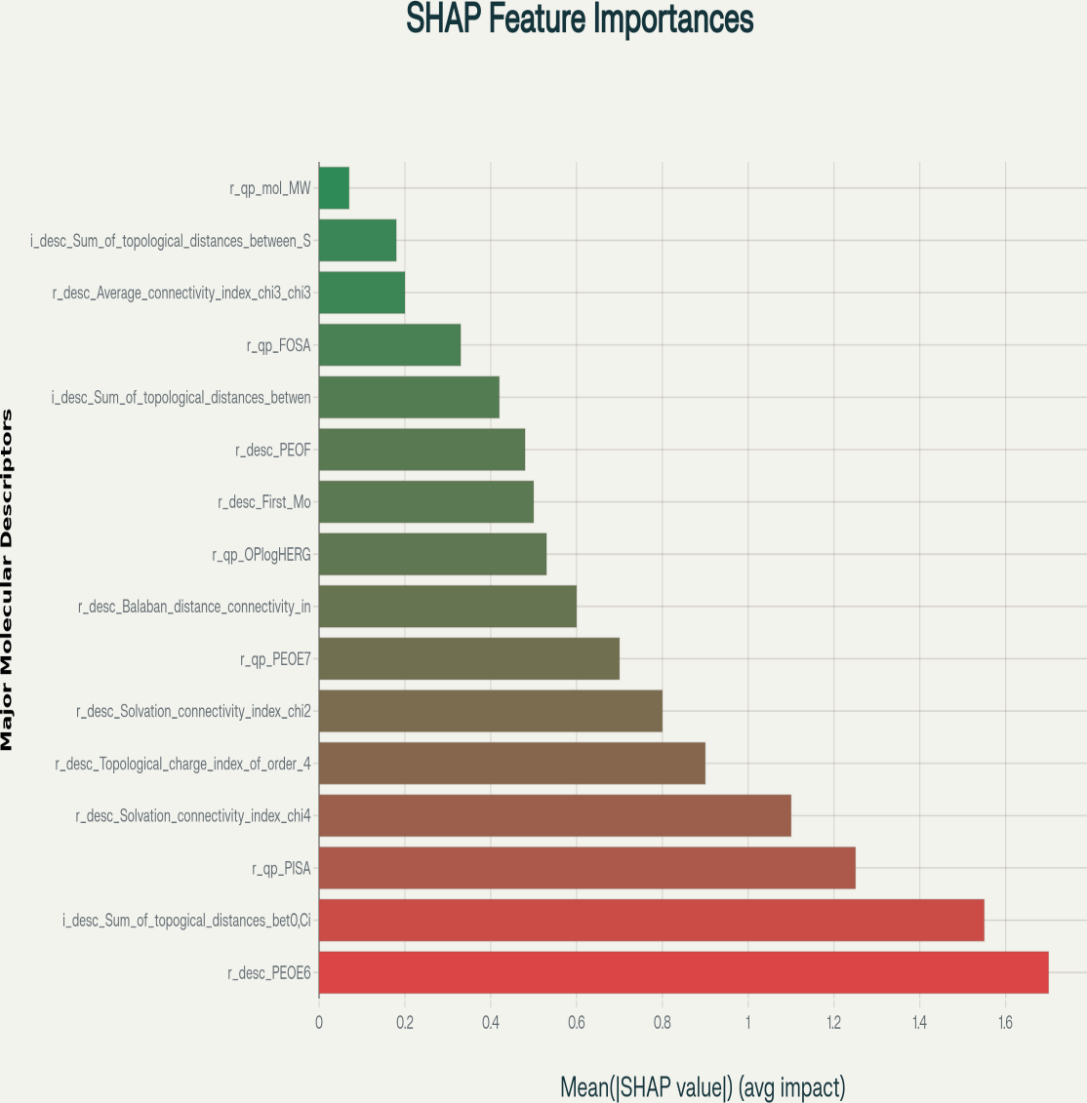
Shapley additive explanations (SHAP) summary plot showing the mean absolute SHAP values of molecular descriptors and their average impact on model predictions for inhibition of DNA polymerase η activity. Higher SHAP values indicate greater importance in predicting compound activity. The most influential descriptors include *r_desc_PEOE6* (electronic properties)*, r_qp_QPlogPC16* (partition coefficients), and *i_desc_Sum_of_topological_distances_between_O.Cl* (topological distances between oxygen and chlorine atoms). Secondary features such as *r_qp_PISA* (polar surface area) and solvation-related descriptors also contribute significantly to the model’s predictions. Lower-ranked descriptors, such as *r_qp_FOSA* (hydrophobic surface area) and r_qp_mol_MW (molecular weight), provide additional structural insights but have less impact on activity than the top-ranked features.

The SHAP summary plot identified r_desc_PEOE6 (electronic properties) as the most influential descriptor, with a mean absolute SHAP value 23% higher than the next best feature (Figure 2).

The second and third top-ranked features were *r_qp_QPlogPC16* (partition coefficients) and *i_desc_Sum_of_topological_distances_between_O.Cl* (atom spacing), respectively. Secondary contributors included r_qp_PISA (polar surface area) and solvation indices such as *r_desc_Solvation_connectivity_index_chi-4*, which stabilized interactions within the polymerase active site. Lower-impact descriptors such as *r_qp_FOSA* (hydrophobic surface area) and *r_qp_mol_MW* (molecular weight) provided structural insights but contributed minimally to predictive reliability.

## Discussion

### Principal Findings

The exceptional predictive performance of ensemble methods, particularly random forest and XGBoost, underscores their suitability for modeling the complex, nonlinear relationships inherent in hpol η inhibition [82-92]. Random forest achieved near-perfect testing metrics (MSE=0.0003; *R*²=0.9998), demonstrating robust generalization through feature space partitioning and aggregation of decision trees. This finding aligns with prior studies in which ensemble methods excelled for biological datasets, such as cancer transcriptome prediction of cell survival, due to their capacity to handle high-dimensional, sparse molecular descriptors [83,85-87]. The minimal performance gap between training and testing (ΔMSE=0.0001 [%]²) highlights effective overfitting mitigation, a critical advantage given the multicollinearity observed in QSAR datasets. XGBoost’s superior performance over neural networks (testing MSE=0.0002 vs 0.0008) further emphasizes gradient-boosted trees’ adaptability to sparse feature spaces, a finding consistent with their success in predicting protein-DNA binding affinity [23,84,92-96]. In contrast, linear models such as lasso regression (testing MSE=0.0032) revealed the necessity of regularization to manage sparsity, although at the cost of predictive accuracy—a trade-off well documented in drug discovery applications [84-93].

SHAP analysis identified electronic properties (r_desc_PEOE6) as the most critical determinant of inhibition activity, with a mean absolute SHAP value 23% higher than the second-ranked descriptor. This aligns with crystallographic evidence showing that charge distribution governs ligand binding stabilization in polymerase active sites [80,97]. The prominence of partition coefficients (r_qp_QPlogPC16) underscores lipophilicity’s dual role in cellular permeability and target engagement, a principle central to antiviral drug design [98,99]. Structural descriptors such as *i_desc_Sum_of_topological_distances_between_O.Cl* further emphasize steric complementarity requirements, mirroring findings in DNA polymerase β inhibition studies where atomic spacing dictated binding specificity [82,100-102]. Secondary features, including polar surface area (*r_qp_PISA*) and solvation indices (*r_desc_Solvation_connectivity_index_chi-4*) [83], elucidate how compounds stabilize aqueous-phase interactions, consistent with enzyme-substrate kinetic models [103-105]. While lower-impact descriptors (*r_qp_FOSA, r_qp_mol_MW*) provided auxiliary structural insights, their minimal contributions suggest prioritization of electronic and topological optimization in rational drug design [106,107].

The models’ consistent error distribution (MAPE: 0.89%‐5.1%) across activity ranges indicates reliability for moderate-activity compounds but exposes limitations in predicting extreme potencies. This mirrors similar challenges observed in solubility modeling, where outlier compounds often defy linear or ensemble-based predictions [108,109]. The clustering of MedAE around 0.01 suggests that while the models capture general trends, they struggle with highly potent inhibitors—a critical gap in drug discovery pipelines. This limitation likely stems from insufficient representation of extreme-activity compounds in training data, a common issue for biochemical datasets. Future work can address this limitation through synthetic minority oversampling or adversarial training techniques.

Methodologically, the integration of SHAP values bridges the interpretability-accuracy divide. While simpler models such as linear regression underperformed by 2 orders of magnitude, SHAP’s ability to deconvolute feature contributions enables actionable insights without sacrificing predictive power [82,83,110]. For instance, the identification of *r_desc_PEOE6* as a top predictor provides a direct optimization target for medicinal chemists: tuning electronic properties to enhance binding affinity. Similarly, *r_qp_QPlogPC16*’s influence offers a pathway to balancing lipophilicity and solubility—a strategy validated in recent hpol η inhibitor development [83]. Integrating molecular-dynamic simulations may enhance predictive accuracy for structurally flexible compounds.

While our models emphasize solvation indices, Salgado et al [111] prioritized hydrogen-bonding descriptors in their polymerase inhibition studies. Discrepancies between these approaches may reflect hpol η’s uniquely hydrophobic active site, suggesting the need for crystallographic validation of descriptor-activity relationships. Conversely, consistency with the solvation models by Gupta et al [112] emphasizes the importance of aqueous-interaction stabilization in enzyme kinetics [104,113-115]. Such contrasts highlight the critical role of target-specific descriptor selection in QSAR workflows.

Translating these findings into drug discovery requires balancing multiparameter optimization. For example, improving *r_desc_PEOE6* (electronic distribution) might conflict with *r_qp_QPlogPC16* (lipophilicity) adjustments, necessitating Pareto front analysis to identify optimal compound profiles. Additionally, the moderate impact of *r_qp_QPlogHERG* (cardiac toxicity risk) implies the necessity for parallel absorption, distribution, metabolism, excretion, and toxicity profiling during lead optimization—a practice increasingly adopted in computational drug design.

This study establishes a predictive framework for hpol η inhibitors by combining ensemble methods (for accuracy) and SHAP analysis (for interpretability). The models prioritize electronic distribution, topological alignment, and solvation properties as critical descriptors, directly guiding rational drug design. The integration of these features underscores the need for multidimensional optimization in QSAR workflows, aligning with modern computational approaches.

### Limitations and Future Directions

This study faced challenges in accurately predicting extreme values, highlighting the need for improved methodologies to address outlier prediction. Additionally, the absence of 3D conformational data limits the ability to model dynamic molecular interactions, which is crucial for capturing the full spectrum of polymerase-targeted binding events. Incorporating such structural information in future models will enhance the realism and predictive power of dynamic interaction analyses.

While SHAP analysis effectively identifies key molecular features, mechanistic interpretations, such as the role of r_desc_PEOE6 in binding pocket interactions, require validation through molecular dynamics simulations. This integration will strengthen the biological relevance of feature importance findings [1-5].

Furthermore, the current model’s applicability domain does not extend to metalloenzyme inhibitors, despite structural similarities among DNA polymerases. Expanding the training set to include these compounds could improve model generalizability and utility across a broader range of enzyme targets. Finally, future studies should explore hybrid modeling architectures that combine ensemble learning methods with graph neural networks. Such approaches may better capture both topological and electronic molecular effects, thereby refining QSAR methodologies for diverse enzyme systems. A key limitation of this study is the relatively small sample size compared to the large number of descriptors, which can increase the risk of overfitting despite the application of robust feature selection, regularization, and algorithmic strategies. Although our SHAP analysis identifies key molecular features, this study does not systematically assess pairs of structurally similar compounds with divergent activities, which is essential for fully evaluating model reliability and understanding potential activity cliffs. Addressing this limitation through focused analyses and validation in future studies will enhance the robustness and interpretability of our QSAR models. While our study claims superiority over linear models, it does not include direct comparisons with recent ML–based QSAR (MLQSAR) approaches, such as deep learning–based models. This limits our ability to fully contextualize our results within the broader scope of state-of-the-art MLQSAR studies. Although the high performance across our algorithms suggests model trustworthiness, future work will address this gap by benchmarking our models against advanced MLQSAR and deep learning methodologies. Additionally, this study evaluated model performance using only an internal 20% test split and did not include external validation with independent datasets or prospective testing. This limits the ability to fully assess the generalizability and real-world applicability of the models. Future work will incorporate validation using independent external datasets and prospective testing to rigorously evaluate model robustness, confirm generalizability, and strengthen confidence in their predictive performance in practical applications. Finally, critical toxicity descriptors (eg, r_qp_QPlogHERG) were identified but not optimized in this study. Future work will optimize these key toxicity descriptors to strengthen absorption, distribution, metabolism, excretion, and toxicity profiling and predictive safety. The current models show reduced performance for outliers and extreme inhibition values, which may impact predictive reliability. Future work will explore strategies such as data augmentation, robust loss functions, and uncertainty estimation to improve the models’ resilience to extreme values and enhance prediction accuracy across the full activity range. While this study achieves strong predictive performance, there is a lack of explicit analysis of the model’s applicability domain and chemical space coverage for novel ITBA analogs. Without thorough assessment of the regions in descriptor space where the model is most reliable, the generalizability to structurally diverse or previously unseen compounds remains uncertain. To address this, future work will incorporate formal applicability domain evaluation, such as leverage and distance-based techniques, to more precisely define the confidence boundaries of predictions and ensure robust extrapolation to new ITBA scaffolds and analogs. This will strengthen the practical utility of the model for prospective inhibitor discovery and design.

### Conclusions

The ML-driven QSAR framework presented in this study overcomes cisplatin resistance challenges by identifying hpol η inhibitors with unprecedented precision. Ensemble methods (random forest and XGBoost) outperformed traditional models, capturing nonlinear relationships between molecular features and activity. SHAP analysis prioritized electronic distribution (*r_desc_PEOE6*), lipophilicity (*r_qp_QPlogPC16*), and structural topology (*i_desc_Sum_of_topological_distances_between_O.Cl*) as critical for efficacy, consistent with biochemical binding principles. While limitations persist in predicting extreme-potency compounds, the study provides actionable strategies to optimize inhibitor design. Future integration of dynamic 4D descriptors, experimental validation, and generative AI could accelerate development of next-generation therapies, revitalizing cisplatin-based treatments for resistant cancers through computationally guided precision.

## Supplementary material

10.2196/77890 Multimedia Appendix 1 Molecular database: molecular database of molecular descriptors representing structural, physicochemical, and quantum properties were calculated using Schrödinger MAESTRO 12.5 software [76]. These included 1D attributes (atom count, molecular weight), 2D features (topological indices, functional groups), 3D characteristics (dipole moment, spatial volume), and 4D properties (HOMO-LUMO energies, electronegativity). A total of 220 descriptors were integrated with experimental inhibition data to enable QSAR modeling.

10.2196/77890 Multimedia Appendix 2 Supplementary information.

## Abbreviations

ADMET: absorption, distribution, metabolism, excretion, and toxicity
AI: artificial intelligence
CCC: concordance correlation coefficient
hpol η: human DNA polymerase η
ITBA: indole thio-barbituric acid
KNN: K-nearest neighbor
LightGBM: light gradient boosting machines
MAE: mean absolute error
MAPE: mean absolute percentage error
MedAE: median absolute error
ML: machine learning
MLQSAR: machine learning–based quantitative structure-activity relationship
MSE: mean square error
NMSE: normalized mean square error
NRMSE: normalized root mean square error
PCC: Pearson correlation coefficient
QSAR: quantitative structure-activity relationship
RMSE: root mean square error
RMSLE: root mean squared logarithmic error
SHAP: Shapley additive explanations
SMAPE: symmetric mean absolute percentage error
SMILES: Simplified Molecular Input Line Entry System
TLS: translesion DNA synthesis
XGBoost: extreme gradient boosting

## References

[1] Zhang C, Xu C, Gao X, Yao Q (2022). Platinum-based drugs for cancer therapy and anti-tumor strategies. Theranostics.

[2] Khan SU, Fatima K, Aisha S, Malik F (2024). Unveiling the mechanisms and challenges of cancer drug resistance. Cell Commun Signal.

[3] Sahoo D, Deb P, Basu T, Bardhan S, Patra S, Sukul PK (2024). Advancements in platinum-based anticancer drug development: a comprehensive review of strategies, discoveries, and future perspectives. Bioorg Med Chem.

[4] Jin SK, Baek KH (2025). Unraveling the role of deubiquitinating enzymes on cisplatin resistance in several cancers. Biochim Biophys Acta Rev Cancer.

[5] Zhong L, Li Y, Xiong L (2021). Small molecules in targeted cancer therapy: advances, challenges, and future perspectives. Sig Transduct Target Ther.

[6] Dasari S, Tchounwou PB (2014). Cisplatin in cancer therapy: molecular mechanisms of action. Eur J Pharmacol.

[7] Anand J, Chiou L, Sciandra C (2023). Roles of trans-lesion synthesis (TLS) DNA polymerases in tumorigenesis and cancer therapy. NAR Cancer.

[8] Li LY, Guan YD, Chen XS, Yang JM, Cheng Y (2021). DNA repair pathways in cancer therapy and resistance. Front Pharmacol.

[9] Maiorano D, El Etri J, Franchet C, Hoffmann JS (2021). Translesion synthesis or repair by specialized dna polymerases limits excessive genomic instability upon replication stress. Int J Mol Sci.

[10] Nayak S, Calvo JA, Cantor SB (2021). Targeting translesion synthesis (TLS) to expose replication gaps, a unique cancer vulnerability. Expert Opin Ther Targets.

[11] Saha P, Mandal T, Talukdar AD (2021). DNA polymerase eta: a potential pharmacological target for cancer therapy. J Cell Physiol.

[12] Berdis AJ (2017). Inhibiting DNA polymerases as a therapeutic intervention against cancer. Front Mol Biosci.

[13] Tomar R, Li S, Egli M, Stone MP (2024). Replication bypass of the *N*-(2-deoxy-d-erythro-pentofuranosyl)-urea DNA lesion by human DNA polymerase η. Biochemistry.

[14] Zafar MK, Maddukuri L, Ketkar A (2018). A small-molecule inhibitor of human DNA polymerase η potentiates the effects of cisplatin in tumor cells. Biochemistry.

[15] Kakraba S, Knisley D (2016). A graph-theoretic model of single point mutations in the cystic fibrosis transmembrane conductance regulator. JBT.

[16] Kakraba S (2021). Drugs That Protect against Protein Aggregation in Neurodegenerative Diseases.

[17] Kakraba S (2015). A Hierarchical Graph for Nucleotide Binding Domain 2.

[18] Netsey EK, Kakraba DS, Naandam SM, Yadem AC, Kakraba DS (2021). A mathematical graph-theoretic model of single point mutations associated with sickle cell anemia disease. J Adv Biotechnol.

[19] Knisley DJ, Knisley JR (2014). Seeing the results of a mutation with a vertex weighted hierarchical graph. BMC Proc.

[20] Knisley DJ, Knisley JR, Herron AC (2013). Graph-theoretic models of mutations in the nucleotide binding domain 1 of the cystic fibrosis transmembrane conductance regulator. Comput Biol J.

[21] Balasubramaniam M, Ayyadevara S, Ganne A (2019). Aggregate interactome based on protein cross-linking interfaces predicts drug targets to limit aggregation in neurodegenerative diseases. iScience.

[22] Netsey EK, Naandam SM, Asante J (2025). Structural and functional impacts of SARS-cov-2 spike protein mutations: insights from predictive modeling and analytics. JMIR Bioinform Biotechnol (Forthcoming).

[23] Yang Z, Zhou H, Srivastav S (2025). Optimizing Parkinson’s disease prediction: a comparative analysis of data aggregation methods using multiple voice recordings via an automated artificial intelligence pipeline. Data (Basel).

[24] Kakraba S, Yadem AC, Abraham KE (2025). Unraveling protein secrets: machine learning unveils novel biologically significant associations among amino acids. Computer Science and Mathematics.

[25] Soares TA, Nunes-Alves A, Mazzolari A, Ruggiu F, Wei GW, Merz K (2022). The (re)-evolution of quantitative structure-activity relationship (QSAR) studies propelled by the surge of machine learning methods. J Chem Inf Model.

[26] Ocana A, Pandiella A, Privat C (2025). Integrating artificial intelligence in drug discovery and early drug development: a transformative approach. Biomark Res.

[27] Odugbemi AI, Nyirenda C, Christoffels A, Egieyeh SA (2024). Artificial intelligence in antidiabetic drug discovery: the advances in QSAR and the prediction of α-glucosidase inhibitors. Comput Struct Biotechnol J.

[28] Schneider A, Hommel G, Blettner M (2010). Linear regression analysis. Dtsch Arztebl Int.

[29] Jarantow SW, Pisors ED, Chiu ML (2023). Introduction to the use of linear and nonlinear regression analysis in quantitative biological assays. Curr Protoc.

[30] Schreiber-Gregory DN (2018). Ridge regression and multicollinearity: an in-depth review. Model Assist Stat Appl.

[31] Rubin J, Mariani L, Smith A, Zee J (2023). Ridge regression for functional form identification of continuous predictors of clinical outcomes in glomerular disease. Glomerular Dis.

[32] Ranstam J, Cook JA (2018). LASSO regression. Br J Surg.

[33] Li Y, Lu F, Yin Y (2022). Applying logistic LASSO regression for the diagnosis of atypical Crohn’s disease. Sci Rep.

[34] Hong C, Xiong Y, Xia J (2024). LASSO-based identification of risk factors and development of a prediction model for sepsis patients. Ther Clin Risk Manag.

[35] Freijeiro‐González L, Febrero‐Bande M, González‐Manteiga W (2022). A critical review of LASSO and its derivatives for variable selection under dependence among covariates. Int Statistical Rev.

[36] Delong Ł, V Wüthrich M (2025). Isotonic regression for variance estimation and its role in mean estimation and model validation. N Am Actuar J.

[37] Deng H, Zhang CH (2020). Isotonic regression in multi-dimensional spaces and graphs. Ann Statist.

[38] Chen C, Cao X, Tian L (2019). Partial least squares regression performs well in MRI-based individualized estimations. Front Neurosci.

[39] Vicente-Gonzalez L, Vicente-Villardon JL (2022). Partial least squares regression for binary responses and its associated biplot representation. Mathematics.

[40] Chen J, Zhang X, Hron K (2021). Partial least squares regression with compositional response variables and covariates. J Appl Stat.

[41] Awad M, Khanna R, Awad M, Khanna R (2015). Efficient Learning Machines: Theories, Concepts, and Applications for Engineers and System Designers.

[42] Montesinos López OA, Montesinos López A, Crossa J, López OA, López AM, Crossa J (2022). Multivariate Statistical Machine Learning Methods for Genomic Prediction.

[43] Zou H, Hastie T (2005). Regularization and variable selection via the elastic net. J R Stat Soc Series B.

[44] De Mol C, De Vito E, Rosasco L (2009). Elastic-net regularization in learning theory. J Complex.

[45] Zhang Z, Lai Z, Xu Y, Shao L, Wu J, Xie GS (2017). Discriminative elastic-net regularized linear regression. IEEE Trans Image Process.

[46] Navada A, Ansari AN, Patil S, Sonkamble BA (2011). Overview of use of decision tree algorithms in machine learning.

[47] Song YY, Lu Y (2015). Decision tree methods: applications for classification and prediction. Shanghai Arch Psychiatry.

[48] Mienye ID, Jere N (2024). A survey of decision trees: concepts, algorithms, and applications. IEEE Access.

[49] Breiman L (2001). Random forests. Mach Learn.

[50] Sun Z, Wang G, Li P, Wang H, Zhang M, Liang X (2024). An improved random forest based on the classification accuracy and correlation measurement of decision trees. Expert Syst Appl.

[51] Cutler A, Cutler DR, Stevens JR, Zhang C, Ma Y (2012). Ensemble Machine Learning: Methods and Applications.

[52] Natekin A, Knoll A (2013). Gradient boosting machines, a tutorial. Front Neurorobot.

[53] Aziz N, Akhir EAP, Aziz IA, Jaafar J, Hasan MH, Abas ANC A study on gradient boosting algorithms for development of AI monitoring and prediction systems.

[54] Boldini D, Grisoni F, Kuhn D, Friedrich L, Sieber SA (2023). Practical guidelines for the use of gradient boosting for molecular property prediction. J Cheminform.

[55] Chen T, Guestrin C, Krishnapuram B, Shah M (2016). Proceedings of the 22nd Acm Sigkdd International Conference on Knowledge Discovery and Data Mining.

[56] Raihan MJ, Khan MAM, Kee SH, Nahid AA (2023). Detection of the chronic kidney disease using XGBoost classifier and explaining the influence of the attributes on the model using SHAP. Sci Rep.

[57] Bentéjac C, Csörgő A, Martínez-Muñoz G (2021). A comparative analysis of gradient boosting algorithms. Artif Intell Rev.

[58] Moore A, Bell M (2022). XGBoost, a novel explainable AI technique, in the prediction of myocardial infarction: a UK biobank cohort study. Clin Med Insights Cardiol.

[59] Zhang Y, Ni M, Zhang C Research and application of adaboost algorithm based on SVM.

[60] Wang R (2012). AdaBoost for feature selection, classification and its relation with SVM, a review. Phys Procedia.

[61] Hancock JT, Khoshgoftaar TM (2020). CatBoost for big data: an interdisciplinary review. J Big Data.

[62] Ibrahim AA, Ridwan RL, Muhammed MM, Abdulaziz RO, Saheed GA (2020). Comparison of the CatBoost classifier with other machine learning methods. Int J Adv Comput Sci Appl.

[63] Uddin S, Haque I, Lu H, Moni MA, Gide E (2022). Comparative performance analysis of K-nearest neighbour (KNN) algorithm and its different variants for disease prediction. Sci Rep.

[64] Halder RK, Uddin MN, Uddin MA, Aryal S, Khraisat A (2024). Enhancing K-nearest neighbor algorithm: a comprehensive review and performance analysis of modifications. J Big Data.

[65] Zhang Z (2016). Introduction to machine learning: k-nearest neighbors. Ann Transl Med.

[66] Guo G, Wang H, Bell D, Bi Y, Greer K, Meersman R, Tari Z, Schmidt DC, Meersman R, Tari Z, Meersman R, Tari Z, Schmidt DC (2003). On The Move to Meaningful Internet Systems 2003: CoopIS, DOA, and ODBASE.

[67] Uhrig RE (1995). Introduction to artificial neural networks.

[68] Han SH, Kim KW, Kim S, Youn YC (2018). Artificial neural network: understanding the basic concepts without mathematics. Dement Neurocognitive Disord.

[69] Schmidhuber J (2015). Deep learning in neural networks: an overview. Neural Netw.

[70] Grossi E, Buscema M (2007). Introduction to artificial neural networks. Eur J Gastroenterol Hepatol.

[71] Goel A, Goel AK, Kumar A (2023). The role of artificial neural network and machine learning in utilizing spatial information. Spat Inf Res.

[72] Deringer VL, Bartók AP, Bernstein N, Wilkins DM, Ceriotti M, Csányi G (2021). Gaussian process regression for materials and molecules. Chem Rev.

[73] Ebden M (2015). Gaussian processes: a quick introduction.

[74] Schulz E, Speekenbrink M, Krause A (2018). A tutorial on gaussian process regression: modelling, exploring, and exploiting functions. J Math Psychol.

[75] Mendelsohn LD (2004). ChemDraw 8 Ultra, Windows and Macintosh versions. J Chem Inf Comput Sci.

[76] Sankar K, Trainor K, Blazer LL (2022). A descriptor set for quantitative structure-property relationship prediction in biologics. Mol Inform.

[77] Sivakumar M, Parthasarathy S, Padmapriya T (2024). Trade-off between training and testing ratio in machine learning for medical image processing. PeerJ Comput Sci.

[78] Shimizu H, Enda K, Shimizu T (2022). Machine learning algorithms: prediction and feature selection for clinical refracture after surgically treated fragility fracture. J Clin Med.

[79] Prokhorenkova L, Gusev G, Vorobev A, Dorogush AV, Gulin A (2018). Catboost: unbiased boosting with categorical features. Adv Neural Inf Process Syst.

[80] Jierula A, Wang S, Oh TM, Wang P (2021). Study on accuracy metrics for evaluating the predictions of damage locations in deep piles using artificial neural networks with acoustic emission data. Appl Sci (Basel).

[81] Van RG, Drake FL (2006). Python reference manual. https://www.python.org/doc/.

[82] Li Z (2022). Extracting spatial effects from machine learning model using local interpretation method: an example of SHAP and XGBoost. Comput Environ Urban Syst.

[83] Wang H, Liang Q, Hancock JT, Khoshgoftaar TM (2024). Feature selection strategies: a comparative analysis of SHAP-value and importance-based methods. J Big Data.

[84] Khan S, Noor S, Javed T (2025). XGBoost-enhanced ensemble model using discriminative hybrid features for the prediction of sumoylation sites. BioData Min.

[85] Vamathevan J, Clark D, Czodrowski P (2019). Applications of machine learning in drug discovery and development. Nat Rev Drug Discov.

[86] Liu M, Srivastava G, Ramanujam J, Brylinski M (2024). Insights from augmented data integration and strong regularization in drug synergy prediction with SynerGNet. Mach Learn Knowl Extr.

[87] Obaido G, Mienye ID, Egbelowo OF (2024). Supervised machine learning in drug discovery and development: algorithms, applications, challenges, and prospects. Machine Learning with Applications.

[88] Sharma A, Lysenko A, Jia S, Boroevich KA, Tsunoda T (2024). Advances in AI and machine learning for predictive medicine. J Hum Genet.

[89] Huang S, Xu Q, Yang G, Ding J, Pei Q (2025). Machine learning for prediction of drug concentrations: application and challenges. Clin Pharmacol Ther.

[90] Patel L, Shukla T, Huang X, Ussery DW, Wang S (2020). Machine learning methods in drug discovery. Molecules.

[91] Ohnuki Y, Akiyama M, Sakakibara Y (2024). Deep learning of multimodal networks with topological regularization for drug repositioning. J Cheminform.

[92] Ahmed NY, Alsanousi WA, Hamid EM (2024). An efficient deep learning approach for DNA-binding proteins classification from primary sequences. Int J Comput Intell Syst.

[93] Thedinga K, Herwig R (2022). A gradient tree boosting and network propagation derived pan-cancer survival network of the tumor microenvironment. iScience.

[94] Claude E, Leclercq M, Thébault P, Droit A, Uricaru R (2024). Optimizing hybrid ensemble feature selection strategies for transcriptomic biomarker discovery in complex diseases. NAR Genomics and Bioinformatics.

[95] Wu BR, Ormazabal Arriagada S, Hsu TC, Lin TW, Lin C (2024). Exploiting common patterns in diverse cancer types via multi-task learning. NPJ Precis Oncol.

[96] Airlangga G, Liu A (2025). A hybrid gradient boosting and neural network model for predicting urban happiness: integrating ensemble learning with deep representation for enhanced accuracy. Mach Learn Knowl Extr.

[97] Arora K, Schlick T (2004). In silico evidence for DNA polymerase-beta’s substrate-induced conformational change. Biophys J.

[98] Jonsson CB, Golden JE, Meibohm B (2021). Time to “Mind the Gap” in novel small molecule drug discovery for direct-acting antivirals for SARS-CoV-2. Curr Opin Virol.

[99] Markowicz-Piasecka M, Markiewicz A, Darłak P (2022). Current chemical, biological, and physiological views in the development of successful brain-targeted pharmaceutics. Neurotherapeutics.

[100] Wang W, Wu EY, Hellinga HW, Beese LS (2012). Structural factors that determine selectivity of a high fidelity DNA polymerase for deoxy-, dideoxy-, and ribonucleotides. Journal of Biological Chemistry.

[101] Beard WA, Wilson SH (2014). Structure and mechanism of DNA polymerase β. Biochemistry.

[102] Batra VK, Beard WA, Shock DD, Pedersen LC, Wilson SH (2008). Structures of DNA polymerase β with active-site mismatches suggest a transient abasic site intermediate during misincorporation. Mol Cell.

[103] Mabesoone MFJ, Palmans ARA, Meijer EW (2020). Solute–solvent interactions in modern physical organic chemistry: supramolecular polymers as a muse. J Am Chem Soc.

[104] Wang S, Meng X, Zhou H, Liu Y, Secundo F, Liu Y (2016). Enzyme stability and activity in non-aqueous reaction systems: a mini review. Catalysts.

[105] Tomasi J, Mennucci B, Cammi R (2005). Quantum mechanical continuum solvation models. Chem Rev.

[106] Senhora FV, Chi H, Zhang Y, Mirabella L, Tang TL, Paulino GH (2022). Machine learning for topology optimization: physics-based learning through an independent training strategy. Comput Methods Appl Mech Eng.

[107] Tang T, Wang L, Zhu M (2024). Topology optimization: a review for structural designs under statics problems. Materials (Basel).

[108] Kazmi B, Taqvi SA, Juchelkov D, Li G, Naqvi SR (2025). Artificial intelligence-enhanced solubility predictions of greenhouse gases in ionic liquids: a review. Results Eng.

[109] Panapitiya G, Girard M, Hollas A (2022). Evaluation of deep learning architectures for aqueous solubility prediction. ACS Omega.

[110] Mohanty PK, Francis SA, Barik RK, Roy DS, Saikia MJ (2024). Leveraging shapley additive explanations for feature selection in ensemble models for diabetes prediction. Bioengineering (Basel).

[111] Salgado PS, Makeyev EV, Butcher SJ, Bamford DH, Stuart DI, Grimes JM (2004). The structural basis for RNA specificity and Ca2+ inhibition of an RNA-dependent RNA polymerase. Structure.

[112] Gupta MN (1992). Enzyme function in organic solvents. Eur J Biochem.

[113] Zeindlhofer V, Schröder C (2018). Computational solvation analysis of biomolecules in aqueous ionic liquid mixtures: from large flexible proteins to small rigid drugs. Biophys Rev.

[114] Warshel A, Aqvist J, Creighton S (1989). Enzymes work by solvation substitution rather than by desolvation. Proc Natl Acad Sci USA.

[115] Sethi A, Agrawal N, Brezovsky J (2024). Impact of water models on the structure and dynamics of enzyme tunnels. Comput Struct Biotechnol J.

