# Supplementary material for: Machine Learning–Enhanced Quantitative Structure-Activity Relationship Modeling for DNA Polymerase Inhibitor Discovery: Algorithm Development and Validation
Source: JMIR AI. 2025 Dec 3;4:e77890. doi: 10.2196/77890 (PMC12675996; doi:10.2196/77890)
Supplement: Multimedia Appendix 2 [file ai-v4-e77890-s002.docx]

Samuel Kakraba^1,2,^*, Srinivas Ayyadevara^3,4^, Aayire C. Yadem^5^, Kuukua E. Abraham^6^, Cesar M. Compadre^7^, and Robert J. Shmookler Reis^3,4, *^

1. Department of Biostatistics and Data Science, Tulane Celia Scott Weatherhead School of Public Health and Tropical Medicine, Tulane University, New Orleans, LA 70112, USA; [skakraba@tulane.edu](mailto:skakraba@tulane.edu) (SK)
2. Tulane Center for Aging, School of Medicine, Tulane University, New Orleans, LA 70112, USA
3. Department of Geriatrics, University of Arkansas for Medical Sciences, Little Rock, AR 72205, USA; AyyadevaraSrinivas@uams.edu (SA); ReisRobertJS@uams.edu (RJSR)
4. Central Arkansas Veterans Healthcare Service, Little Rock, AR 72205, USA
5. CytoAstra LLC, Little Rock, AR 72211, USA
6. Department of Mathematics, Woodale High School, 5151 Scottdale Ave., Memphis, TN 38118, USA; abrahamk1@scsk12.org (KEA)
7. Department of Pharmaceutical Sciences, University of Arkansas for Medical Sciences, Little Rock, AR 72205, USA; CMCompadre@uams.edu (CMC)

**^*^**Correspondence: SK, skakraba@tulane.edu, Tel.:+1- 504-988-2475

^*^RJSR, rjsr@uams.edu, Tel.: +1-501-257-5560

**Supplementary Material S1: Molecular Database Construction and Integration**

**1. Molecular Database Generation**

**1.1 Compound Curation**
The dataset comprised 85 indole thio-barbituric acid (ITBA) analogs with experimentally validated human DNA polymerase η (hpol η) inhibition activity, quantified as mean percentage activity reduction. Six compounds (e.g., PNR-7-02) were excluded due to unreported hpol η activity. Three outliers (PNR-5-88, PNR-3-50, PNR-3-64) were identified via scatter plots and interquartile range (IQR) analysis and removed to ensure dataset integrity.

**1.2 Structural Preparation**

- **Chemical Structure Drafting**: Initial structures were designed in ChemDraw, converted to SMILES format, and then to SYBYL Mol2 files using Schrödinger MAESTRO for 3D visualization.
- **Energy Minimization**: Optimized molecular geometries using the OPLS4 force field to ensure stable conformers.
- **Alignment**: Conserved ITBA cores were structurally aligned to standardize side-chain modifications, ensuring consistent descriptor computation.

**1.3 Molecular Descriptor Computation**
Using Schrödinger MAESTRO, 220 molecular descriptors spanning four dimensions were calculated:

- **1D**: Atom count, molecular weight, logP.
- **2D**: Topological indices (e.g., Balaban distance connectivity index), functional groups.
- **3D**: Dipole moment, spatial volume, HOMO-LUMO energies.
- **4D**: Solvation connectivity indices, polar surface area (PSA), and electronegativity-weighted charge indices.

Quantum chemical descriptors (e.g., HOMO energy, polarizability) were derived to capture electronic behavior during polymerase interactions.

**2. Data Preprocessing**

**2.1 Activity Normalization**
Inhibition activity values were standardized to a 0–100% scale, with lower values indicating stronger inhibition.

**2.2 Feature Engineering**

- **Missing Data**: Compounds with incomplete descriptor profiles were excluded.
- **Normalization**: Features were scaled using StandardScaler (mean = 0, variance = 1) to ensure equal weighting during machine learning (ML) model training.
- **Split**: The dataset was divided into 80% training (68 compounds) and 20% testing (17 compounds) sets using stratified sampling to preserve activity distribution.

**3. Database Structure**
The final molecular database (provided as Dna-polymerase_activity_outliers_3_deleted.csv) includes:

- **Rows**: 82 compounds (after outlier removal).
- **Columns**:
  - **Identifier**: Compound IDs (e.g., PNR-6-89).
  - **Activity**: Mean percentage hpol η inhibition.
  - **Descriptors**: 300+ molecular features (e.g., r_desc_PEOE6, r_qp_QPlogPC16).

Key descriptors identified via SHAP analysis as critical for inhibition include:

1. **Electronic properties**: r_desc_PEOE6 (partial charge distribution).
2. **Lipophilicity**: r_qp_QPlogPC16 (partition coefficient).
3. **Topological distances**: i_desc_Sum_of_topological_distances_between_O.Cl.

**4. Database Integration**

**4.1 Accessibility**
The database is hosted on [repository name/link] in two formats:

- **CSV**: For compatibility with Python/R workflows.
- **.Rda**: For seamless integration with R-based QSAR pipelines.

**4.2 Usage Notes**

- **File Loading**:

import pandas as pd
df = pd.read_csv("Curated molecular database for QSAR.csv")

- **Descriptor Selection**: Prioritize SHAP-validated features (see Section 3) for model interpretability.
- **Updates**: Future versions will include dynamic 4D descriptors (e.g., molecular dynamics trajectories).

**5. Validation**
The database’s robustness was confirmed through:

- **ML Performance**: Random Forest achieved testing MSE = 0.0003, R² = 0.9998.
- **SHAP Consistency**: Top descriptors aligned with known biochemical principles (e.g., electronic properties govern binding affinity).

This database provides a foundation for AI-driven inhibitor discovery and reproducibility in chemoresistance research.

**References:**

Kakraba, S., Drugs That Protect Against Protein Aggregation in Neurodegenerative Diseases. 2021.

Zafar, M.K., et al., A Small-Molecule Inhibitor of Human DNA Polymerase η. *Biochemistry*, 2018.

Sankar, K., et al., A Descriptor Set for QSPR Prediction in Biologics. *Molecular Informatics*, 2022. Sivakumar, M., et al., Trade-off Between Training and Testing Ratios in ML. *PeerJ Comput Sci*, 2024.
